# Supplementary material for: Convolutional neural networks improve species distribution modelling by capturing the spatial structure of the environment
Source: PLoS Comput Biol. 2021 Apr 19;17(4):e1008856. doi: 10.1371/journal.pcbi.1008856 (PMC8084334; doi:10.1371/journal.pcbi.1008856)
Supplement: S1 Protocole — Detailed dataset construction protocole. (PDF) [file pcbi.1008856.s001.pdf]

Detailed dataset construction protocole

Our study is based on a large presence-only dataset built from the Global Biodiversity Informatics Facility (GBIF):

1. Data aggregation: we used the GBIF API[1] to download all occurrences having a GPS coordinates in the French territory[2].

2. Data filtering: we kept only occurrences of species belonging to the *Planteae* kingdom. However, many occurrences are voluntary quantized on a fixed spatial grid to prevent end-users of the platform recovering the exact position of the observed specimens. Whereas this may be justified from a biodiversity conservation perspective, this clearly introduces strong statistical biases. Species observed in the same arbitrary quadrat (up to 10 km) may actually appear as being observed exactly at the same site. This is problematic for the training and testing set separation because if we randomly split occurrences some test occurrences will have the same input than train occurrences and if we process a spatial split we introduce spacial bias between train and test. We thus decided to keep only one occurrence at each distinct location appearing in the raw set of occurrences. In our case, as we work with join species distribution models, we kept only one occurrence per site all species combined.

3. Taxonomic information: We selected occurrence data for taxa identified at the species level or below. We merged infrataxa at corresponding species level. We used the TAXREF taxonomic reference from INPN (French National Inventory of Natural Patrimony)[3], i.e., we assigned synonym taxa to corresponding accepted name, and removed taxa that did not match with a valid name.

[1] `http://api.gbif.org/v1/`

[2] `GBIF.org` (31 October 2018) GBIF Occurrence Download `https://doi.org/10.15468/dl.l4ofpm`

[3] TAXREF v12.0 `https://inpn.mnhn.fr/telechargement/referentielEspece/taxref/12.0/menu`
