# Supplementary material for: Convolutional neural networks improve species distribution modelling by capturing the spatial structure of the environment
Source: PLoS Comput Biol. 2021 Apr 19;17(4):e1008856. doi: 10.1371/journal.pcbi.1008856 (PMC8084334; doi:10.1371/journal.pcbi.1008856)
Supplement: Attachment — Submitted filename: Response_to_reviewers.pdf [file pcbi.1008856.s002.pdf]

Dear Editor, dear reviewers,

We are pleased to submit a new version of our manuscript entitled "Convolutional neural networks improve species distribution modelling by capturing the spatial structure of the environment", to be considered for publication as a Research Article in Plos Computational Biology.

We would like to thank both reviewers for their constructive feedback, which allowed us to greatly improve the quality of our manuscript. We have consistently revised its content to respond to each of the remarks and suggestions. In particular:

- we have clarified the contributions of our study with respect to previous work on deep SDMs
- we conducted additional experiments using classical metrics for SDMs (AUC & TSS)
- we included a discussion about the limitations of these metrics for the type of models we are studying and the reasons why the primary evaluation metric we are using is more adapted
- we have thoroughly reworked the text and the presentation to make them clearer.

In the remainder of this letter we respond in detail to each of the reviewers' comments.

Sincerely,
Benjamin Deneu, on behalf of the authors

Reviewer #1: This paper describes a deep learning approach to species distribution prediction that uses spatial environmental information as input to a convolutional neural network (CNN). The study is well carried out with ablation studies and comparison to other methods. The two main problems with the paper are

1) the exposition could be more clear (see below)(it is suggested that the authors work more on the writing (perhaps consult a native English speaker))

We have consistently revised and improved the style and the clarity of the text.

2) other works [19,21] have already applied CNNs to this problem. (it should be made more clear what the novelty is of this work.)

The main objective of this study is not to demonstrate once again that CNNs can score similarly or better than other approaches, but to understand why and how this new type of model conveys important insights into species distribution dynamics. In particular, we found that the landscape structure captured by these models crucially contributes to improve predictive performance. Moreover, we show that the prediction

gain is particularly improved for rare species, which open promising perspectives for biodiversity monitoring and conservation strategies.

Minor comments:

1. the later → the latter, fixed
2. Define punctual model properly first time used. fixed
3. For practical reasons 6 (data required), most Species Distribution Models (SDMs) are correlative methods 7 relating known species occurrence data to potential environmental predictors [2–7]. 8 Popular examples of such methods include MAXENT [8–10], random forest [11] and 9 boosted regression trees [12–14].

   MaxEnt etc are general regression method that can be applied to SDM. The text has been corrected to point out that MaxEnt, random forest are indeed examples of correlative methods.
4. Eq (1) needs more explanation. Eq (1) had indeed nothing to do here. It was an idea for a new metric based on entropy that was not retained in the final evaluations.
5. The paragraph Predictions is unclear. What is exactly being predicted? I understand a softmax is being used. So it is multinomial classification. But the data is presumably occurrence of each species. What is the conversion here?

   We entirely revised this paragraph to make it clearer and we also revised the structure of the related sections. The presentation of the type of SDM models we are studying is now at the beginning of section "Species Distribution Models".
6. There are also other examples similar to 4. and 5. later in the paper in the same spirit that it should be possible for the authors to spot without be pointed to by a referee. ;-)

   We have carefully double-checked this new version of the document.

Reviewer #2: In this paper, the authors compared convolutional neural networks (CNNs), deep but non-convolutional neural network models (DNNs), boosted trees (BT) and Random Forest (RF) for predicting species distributions. They found that CNNs outperformed the other models for rare species. This demonstrated the usefulness of CNNs.

The authors used top-k accuracy to characterize the performance of the models. However, this approach of model evaluation is difficult for species distribution modelling practitioners to understand because they always use the conventional model accuracy measures (including the area under the receiver's operating characteristic curve, true skill statistic, sensitivity and specificity, etc.). It's better also to give the model evaluation using these measures.

We re-evaluated the model following the suggested more usual metrics including AUC and true skill statistic. The main limitation of using these methods in our case is the

dynamic of our dataset. The majority of the 4520 species evaluated have very few occurrences in the test set making individual evaluations of AUC and TSS difficult and statistically insignificant. The choice of pseudo-absence data is also an important point and can arbitrarily influence the score obtained (it is a well known bias of these evaluation metrics). The simplest choice would be to randomly pick the pseudo-absence from the test set occurrences of the other species, but the most frequent species would then be over-represented. As these species are often those with the widest or least specific distributions, this choice would penalize the evaluation of other species that may share certain environments where they are present. To avoid this bias, we made a uniform draw among the other species, the occurrences of the chosen species then serve as pseudo-absences. We included a thorough discussion about these concerns in the paper to better justify the use of the top-k accuracy. We are aware that top-k accuracy is also not perfect either because the size of the set of species observable at a given location can be very variable. Therefore, in future work, we plan to work on adaptive set-valued accuracy metrics. However, this was not the scope of this paper.

Specifics:

- P2 L71: Table ?? (?) fixed
- P7 L193: "is apply at" may be changed to "is applied at"? fixed
- P8 L214: "are uses" may be changed to "are used"? fixed
- P10 L298-299: "the environmental neighborhood more than the punctual environment matters for prediction"? fixed
- P10 L309: "it's"? fixed
- P11 L356: change "parcimonious" to "parsimonious" fixed
- Some information is lost for this reference: 52. Botella C, Joly A, Monestiez P, Munoz F, Bonnet P. Bias in presence-only niche models related to sampling effort and species niches: lessons for background point selection. 2020;. fixed
